# Overexpression of USP39 predicts poor prognosis and promotes tumorigenesis of prostate cancer via promoting EGFR mRNA maturation and transcription elongation

**DOI:** 10.18632/oncotarget.7882

**Published:** 2016-03-03

**Authors:** Yi Huang, Xiu-Wu Pan, Lin Li, Lu Chen, Xi Liu, Jian-Lei Lu, Xiao-Mei Zhu, Hai Huang, Qi-Wei Yang, Jian-Qing Ye, Si-Shun Gan, Lin-Hui Wang, Yi Hong, Dan-Feng Xu, Xin-Gang Cui

**Affiliations:** ^1^ Department of Urinary Surgery of Changzheng Hospital, Second Military Medical University, Shanghai, 200003, China; ^2^ The 2nd Department of Oncology of Chenggong Hospital, Xiamen University, Xiamen, 361003, China; ^3^ Department of Urinary Surgery of Third Affiliated Hospital, Second Military Medical University, Shanghai, 201805, China; ^4^ R & D Departmemt of DuruoBiotechnologies Inc., Shanghai, 200233, China; ^5^ Department of Urinary Surgery of Ruijin Hospital, Shanghai Jiaotong University, Shanghai, 200025, China

**Keywords:** USP39, prostate cancer, prognosis, tumorigenesis, EGFR

## Abstract

Castration resistance is a serious problem facing clinical treatment of prostate cancer (PCa). The underlying molecular mechanisms of acquired proliferation ability of tumor cells upon androgen deprivation are largely undetermined. In the present study, we identified that ubiquitin specific peptidase 39 (USP39) was significantly upregulated in PCa samples and cell lines. Elevated USP39 expression was positively correlated with Gleason score, predicted a poor outcome, and functioned as an independent risk factor for biochemical recurrence (BCR) especially in patients with a Gleason score ≤7. Our cell-based study showed that the expression level of USP39 was the highest in AR-negative PCa cell lines. Knockdown of USP39 in PCa cells inhibited cancer colony formation and tumor cell growth, and induced G2/M arrest and cell apoptosis. Microarray analysis suggested that knockdown of USP39 caused a reduced expression of EGFR. Silencing of USP39 inhibited the expression of EGFR 3′-end, and presented a remarkable block to the maturation of EGFR mRNA, suggesting that silencing of USP39 decreased the transcriptional elongation and maturation of EGFR mRNA. Oncomine datasets analysis showed that USP39 expression was positively correlated with EGFR level. The above findings suggest that USP39 plays a vital oncogenic role in the tumorigenesis of PCa and may prove to be a potential biomarker for predicting the prognosis of PCa patients.

## INTRODUCTION

Prostate cancer (PCa) is the second most commonly diagnosed malignancy in men worldwide [1]. More than 25-50% patients are diagnosed as biochemical recurrence (BCR) after radical prostatectomy [2]. These patients are at a higher risk for distant metastasis and PCa-specific death [3]. Androgen receptor (AR) signaling pathway is the primary mediator of PCa. Although androgen deprivation therapy (ADT) is a widely used therapy for patients with metastatic prostate cancer (mPCa), almost all patients with mPCa progress to castration resistant prostate cancer (CRPC) in the late stage. How PCa acquires proliferative ability upon androgen deprivation remains unclear. The clinicopathological features including Gleason score or pT are usually used to predict the BCR of PCa patients after radical prostatectomy. However, these indexes cannot fully reflect the divergent disease courses [4]. Therefore, it is necessary to study the molecular mechanisms of PCa, and to screen out novel biomarkers for prognostic prediction of PCa patients.

Ubiquitin-specific peptidase (USP39) is a member of the deubiquitylation family [5], however its biological function remains unclear. Studies have shown that USP39 is fully deprived of de-ubiquitinating activity [6, 7]. The USP39 ortholog of the yeast protein Sad1p was found to function in two ways: assembly of U4 snRNP into U6 snRNP, and pre-mRNA splicing [7]. Yesenia Ríos et al [8] reported that USP39 mutant down-regulated rb1 through inducing splicing defect by removing the intron between exon 3 and exon 4. Therefore, USP39 is considered to belong to the spliceosome family.

Spliceosome, containing over 150 distinct proteins and 5 small nuclear (sn) RNAs, is a complex of intracellular protein/RNA that guides splicing of pre-mRNA in eukaryotic cells [9]. Abnormal expressions of spliceosome proteins have been reported to be associated with tumorigenesis. SF3B1, one of the spliceosome proteins, is mutated in multiple malignant tumors, such as myelodysplastic syndromes and lymphocytic leukemia [10, 11]. Sam68 is involved in splicing of CCND1 mRNA [12] to promote the proliferation and survival of PCa cells [13]. A recent study identifies that SND1, a cofactor of Sam68, was highly expressed in PCa cells and promotes cell proliferation by promotingCD44 splicing [14].

USP39 is a conserved protein, known as Sad1p in yeast and a 65 kDa (65K) SR-related protein in humans [7, 15]. Both proteins are involved in the assembly of the mature spliceosome complex [7, 15]. In addition, USP39 is also required to maintain the spindle checkpoint and support successful cytokinesis [6]. Silencing of USP39 could specifically reduce the Aurora B mRNA expression [6], and mutation of zebrafish USP39 induces rb1 splicing defects and pituitary lineage expansion [8]. Some more recent studies have reported the involvement of USP39 in malignant cell growth. Wang et al. [16] have reported that USP39is overexpressed in breast cancer, and is involved in the tumorigenesis. Wen et al. [17] have reported that USP39 is a target of SUMOylation specific protein (SENP) in the proliferation of PCa cells. However, the clinical relevance of USP39 in PCa and its molecular mechanism have rarely been reported.

The objective of this study was to investigate the expression pattern of USP39 in PCa tissue by immunohistochemical staining and Oncomine data-mining, to identify the molecular target of USP39 in PCa cell lines, and to uncover the underlying regulatory mechanism, in an attempt to gain novel insights into tumorigenesis of PCa.

## RESULTS

### USP39 is overexpressed in prostate cancer tissues and is highly correlated to Gleason score

**Figure 1:**
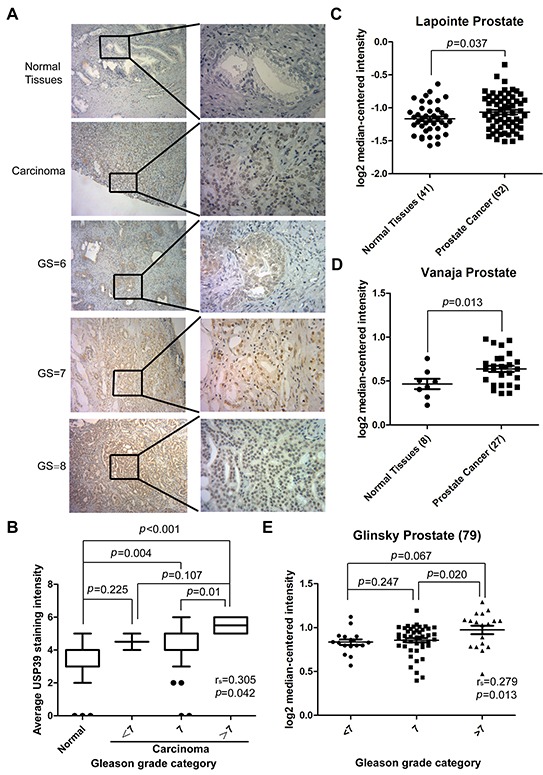
USP39 is overexpressed in PCa tissues and positively correlated with Gleason score **A/B.** Representative immunohistochemical images are presented to show USP39 expression in PCa and paired normal tissues. The data show that patients with increased USP39 hadhigh Gleason scores. **C/D.** Two ONCOMINE microarray datasets of PCa (Lapointe.el and Vanaja.el) were analyzed between cancer and normal tissues, and the *P* value was 0.037 and 0.013, respectively. **E.** Glinsky's data indicate a correlation between USP39 expression and Gleason score. (*P* = 0.013)

PCa specimens were analyzed for USP39 expression by immunohistochemical staining. The characteristics of the patients are shown in Table 1. The result showed high expression levels of USP39 in PCa tissue and strong nuclear staining in PCa cells (Figure 1A). In PCa group, 64.86% of the specimens presented strong positive *vs.* 24.32% in the normal group (*P*=0.001) (Table 2). As shown in Figure 1C and 1D, two datasets of oncomine also revealed that USP39 expression level in PCa tissue was elevated compared tonormal tissues by using its value of log2 median-center intensity (Lapointe prostate: *P*=0.039; Vanaja prostate: *P*=0.013). Moreover, the expression level of USP39 was positively correlated with Gleason score (spearman's correlation=0.305, *P*=0.042), and significantly higher in PCa tissues with a Gleason score greater than 7 (Gleason grade=7 *vs.* >7: *P*=0.01) (Figure 1B). The Glinsky prostate dataset from ONCOMINE showed the same result (Spearman's correlation=0.279,*P*=0.013, Gleason score=7 *vs.*>7:*P*=0.020) (Figure 1E).

**Table 1:** Characteristics of patients by the positive expression of USP39

	−∼+	++	+++	*p*
Basic characteristics	4	11	30	
Mean Age, yr (range)	74 (68-80)	70(57-80)	67(46-82)	0.352^a^
PSA Level: n (%)				0.105^a^
<10ng/mL	2(50.0)	5(45.5)	9(30.0)	
10-20ng/mL	2(50.0)	2(18.2)	4(13.3)	
>20ng/mL	0	4(36.4)	17(56.7)	
Gleason Score (ISUP): n (%)				0.300^a^
3+3	0	1(9.1)	1(3.3)	
+4	2(50.0)	4(36.4)	11(36.7)	
4+3	2(50.0)	6(54.5)	10(33.3)	
4+4	0	0	6(20.0)	
4+5	0	0	2(6.7)	
T stage: n (%)				0.268^a^
≤T2a	4(100.0)	10(90.9)	22(73.3)	
T2b	0	1(9.1)	7(23.3)	
≥T2c	0	0	1(3.3)	
Capsular Invasion: n (%)				0.334^b^
No	4(100.0)	11(100.0)	26(86.7)	
Yes	0	0	4(13.3)	
Seminal Vesicle Invasion: n (%)				0.584^b^
No	4(100.0)	10(90.9)	25(83.3)	
Yes	0	1(9.1)	5(16.7)	
Perineural Invasion: n (%)				0.126^b^
No	4(100.0)	11(100.0)	23(76.7)	
Yes	0	0	7(23.3)	

a Kruskal-Wallis test

b chi-square test

**Table 2:** IHC staining of USP39 expression in prostate cancer tissues

	−∼+	++	+++	Case	The rate of strong positive	*p*
Carcinoma	2(5.4)	11(29.7)	24(64.9)	37	64.86%	0.001^a^
Paracarcinoma	3(8.1)	25(67.6)	9(24.3)	37	24.32%	

a Mann-Whitney U test

### Overexpression of USP39 predicts poor prognosis in PCa patients

**Figure 2:**
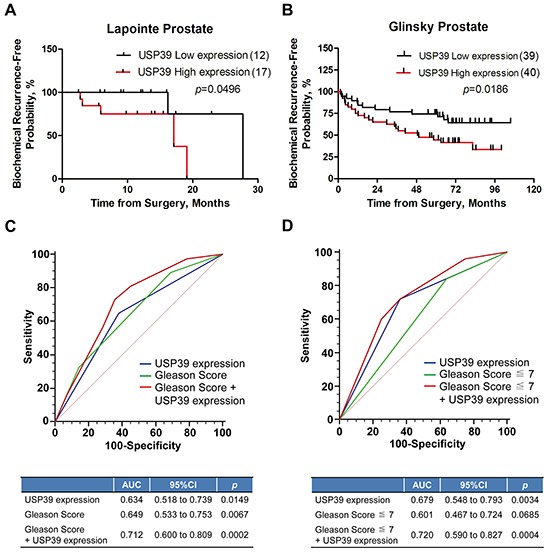
Kaplan-Meier analysis of USP39 is highly relevant to the poor prognosis of PCa patients **A/B.** The correlation between USP39 expression and BCR free survival was analyzed using Lapointe and Glinsky Prostate datasets (ONCOMINE). **C/D.** ROC curve was used to analyze the prediction ability of USP39, Gleason score, and combination of the two factors.

BCR of PCa patients was analyzed using oncomine datasets. Glinsky dataset (Table 3) indicated that overexpression of USP39 was positively correlated with Gleason score (*P*=0.029) and BCR (*P*=0.018). Kaplan-Meier survival analysis showed that elevated expression of USP39 suppressed the BCR free survival in both Lapointe and Glinsky datasets (*P*=0.0496 and *P*=0.0186, respectively) (Figure 2A and 2B).

**Table 3:** USP39 expression and patient characteristics of Glinsky prostate dataset

	No. Pts	No. USP39		*p* Value
		Low	High	
Overall: n (%)	79(100.0)	39(49.4)	40(50.6)	
Mean patient age, y (range)	60.6(44.9-72.7)	59.6(44.9-68.9)	61.7(50.1-72.7)	0.131^a^
PSA Level: n (%)				0.790^b^
<4	6(7.6)	3(7.7)	3(7.5)	
4-10	44((55.7)	21(53.8)	23(57.5)	
>10	29(36.7)	15(38.5)	14(35.0)	
Gleason Score: n (%)				0.029^b^
<7	17(21.5)	12(30.8)	5(12.5)	
7	44(55.7)	21(53.8)	23(57.5)	
>7	18(22.8)	6(15.4)	12(30.0)	
N stage: n (%)				0.248^c^
N0	76(96.2)	39(100.0)	37(92.5)	
N1+	3(3.8)	0	3(7.5)	
T stage: n (%)				0.973^b^
≤T2a	50(63.3)	25(64.1)	25(62.5)	
T2b	20(25.3)	9(23.1)	11(27.5)	
≥T2c	9(11.4)	5(12.8)	4(10.0)	
Capsular Invasion: n (%)				0.152^c^
None	17(21.5)	8(20.5)	9(22.5)	
Focal	6(7.6)	3(7.7)	3(7.5)	
Invasive	18(22.8)	13(33.3)	5(12.5)	
Established	38(48.1)	15(38.5)	23(57.5)	
Recurrence Status: n (%)				0.018^c^
NO	42(53.2)	26(66.7)	16(40.0)	
YES	37(46.8)	13(33.3)	24(60.0)	

a T-test

b Wilcoxon rank sum test

c chi-square test

We also performed univariate and multivariate Cox regression analyses of BCR using Glinsky PCa dataset (Table 4). Univariate analysis indicated that high USP39 expression (Hazard ratio (HR) = 2.167,*P*<0.025), Gleason score>7 (HR=1.966, *P*=0.001), high PSA (HR=1.925, *P*=0.048) and capsular invasion (HR = 6.821, *P*=0.008) were independent risk factors for BCR of prostate cancer patients. Multivariate analysis suggested that capsular invasion (HR = 6.821, *P*= 0.008) and high USP39 expression (HR = 2.167, *P*< 0.025) were hazard factors for predicting BCR in PCa patients.

**Table 4:** Univariate and multivariate Cox regression analysis of BCR of Glinsky prostate dataset

	Univariate COX Regression		Multivariate Cox Regression	
	HR (95% CI)	*p* Value	HR (95% CI)	*p* Value
Age^*^, years		0.086		0.743
<61.2	1.00 (referent)		1.00 (referent)	
≥61.2	1.791(0.921-3.482)		1.130(0.545-2.343)	
PSA Level		0.048		0.238
<10	1.00 (referent)		1.00 (referent)	
≥10	1.925(1.005-3.685)		1.571(0.741-3.331)	
Gleason Score		0.001		0.158
≤7	1.00 (referent)		1.00 (referent)	
>7	1.966(1.516-5.802)		1.774(0.801-3.930)	
N stage		0.081		0.428
N0	1.00 (referent)		1.00 (referent)	
N1+	2.888(0.877-9.515)		1.700(0.457-6.314)	
T stage		0.657		0.118
≤T2b	1.00 (referent)		1.00 (referent)	
≥T2c	1.238(0.481-3.187)		2.268(0.812-6.339)	
Capsular Invasion		0.008		0.017
No	1.00 (referent)		1.00 (referent)	
Yes	6.821(1.638-28.407)		6.142(1.391-27.125)	
USP39 expression		0.025		0.029
Low	1.00 (referent)		1.00 (referent)	
High	2.167(1.100-4.269)		2.240(1.087-4.614)	

* Divided at median

Then, we used ROC curve to analyze the predictive ability of Gleason score and USP39 for BCR and found that Gleason score and USP39 could be used as prognostic factors for BCR of prostate cancer. Analysis of the prediction ability of Gleason score by ROC curve in the Gleason score ≤7 subgroup, showed that Gleason score lost its prediction ability (*P*=0.0685), and the prediction ability of USP39 was higher than that of Gleason score (*P*=0.0034) (Figure 2D). The combination of USP39 and Gleason score showed a better predictive ability (Figure 2C and 2D). Taken together, these results indicated that USP39 might be a biomarker to predict the BCR of postoperative PCa patients, especially when Gleason score was ≤7.

### Lentivirus-mediated efficient knockdown of USP39 gene in PCa cell lines

**Figure 3:**
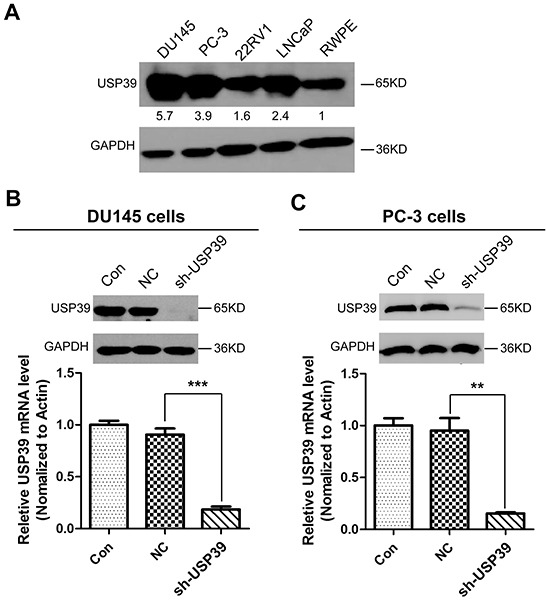
USP39 expression in PCa cell lines and the silencing efficiency of lentivirus-mediated USP39-shRNA **A.** The expression levels of USP39 were detected by Western blotting among PCa cell lines. **B/C.** The knockdown efficiency of USP39 was assessed by qPCR and western blot analysis in DU145 and PC-3 cells. And *P* value is 0.0004 and 0.0073 respectively.

To further investigate the function of USP39, we first compared USP39 expression between normal prostate cell (RWPE) and PCa cell lines including DU145, PC-3, 22RV1, and LNCaP by Western blotting. As shown in Figure 3A, USP39 was up-regulated in PCa cell lines compared to RWPE prostate cell, and especially overexpressed in DU145 and PC-3 androgen independent cell lines (androgen receptor, AR-). Therefore, DU145 and PC-3 with the highest level of USP39 expression were subjected to analysis for lentivirus mediated gene knockdown. DU145 and PC-3 cells with a transduction efficiency of about 90% as represented by the percentage of GFP-expression cells were subjected to functional analysis (Supplementary Figure S1A). Western blot and qRT-PCR indicated an efficient knockdown of USP39 in both cell lines (Figure 3B-3C).

### Silencing of USP39 inhibits proliferation and colony formation by inducing G2/M arrest and apoptosis in PC-3 and DU145 cells

**Figure 4:**
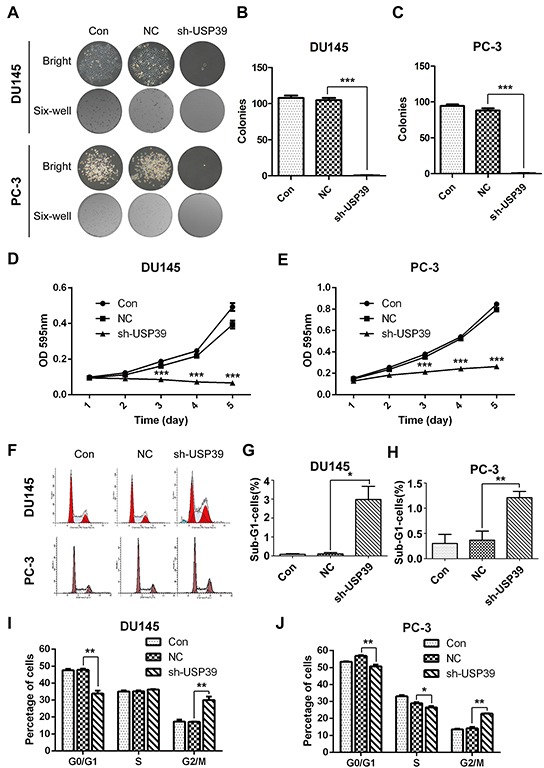
USP39 silencing inhibited cell proliferation and colony formation by inducing of G2/M arrest and apoptosis and in PC-3 and DU145 **A, B.** and **C.** Clonysizes of PC-3 and DU145 were significantly suppressed after USP39 knockdown (*P*=0.0002 and *P*=0.0003, respectively). **D.** and **E.** The proliferation of PC-3 and DU145 was obviously decreased after USP39 silencing (DU145,D2-D5,*P*<0.0001;PC-3,D2-D5,*P*<0.0001). **F, I.** and **J.** USP39 knockdown inducedG2/M arrest in PC-3 and DU145. **G.** and **H.** USP39 silencing induced apoptosis as represented by Sub-G1 accumulation (*p*=0.0039 and *p*=0.0108).

Compared with the control group, plate colony formation assay showed that the sizes of colony and the number of colonies were significantly reduced in USP39 knockdown PC-3 and DU145 cells (Figure 4A-4C), indicating that silencing of USP39 inhibited the colony formation ability of PCa cells. MTT assay showed that USP39 knockdown inhibited cell proliferation. The cell proliferative of PC-3 and DU145 cells was observably decreased after sh-USP39 transduction (*P*<0.001) (Figure 4D-4E). In addition, FACS was performed to evaluate cell cycle distribution and assess the underlying mechanism of proliferation inhibition. It was found that USP39 silencing induced G2/M phase arrest and apoptosis (Figure 4F-4J), implying that the growth inhibition might be associated with increased cell cycle arrest and apoptosis. These results strongly support that USP39 plays an important role in the malignant proliferation of PCa.

### EGFR is a downstream target of USP39

**Figure 5:**
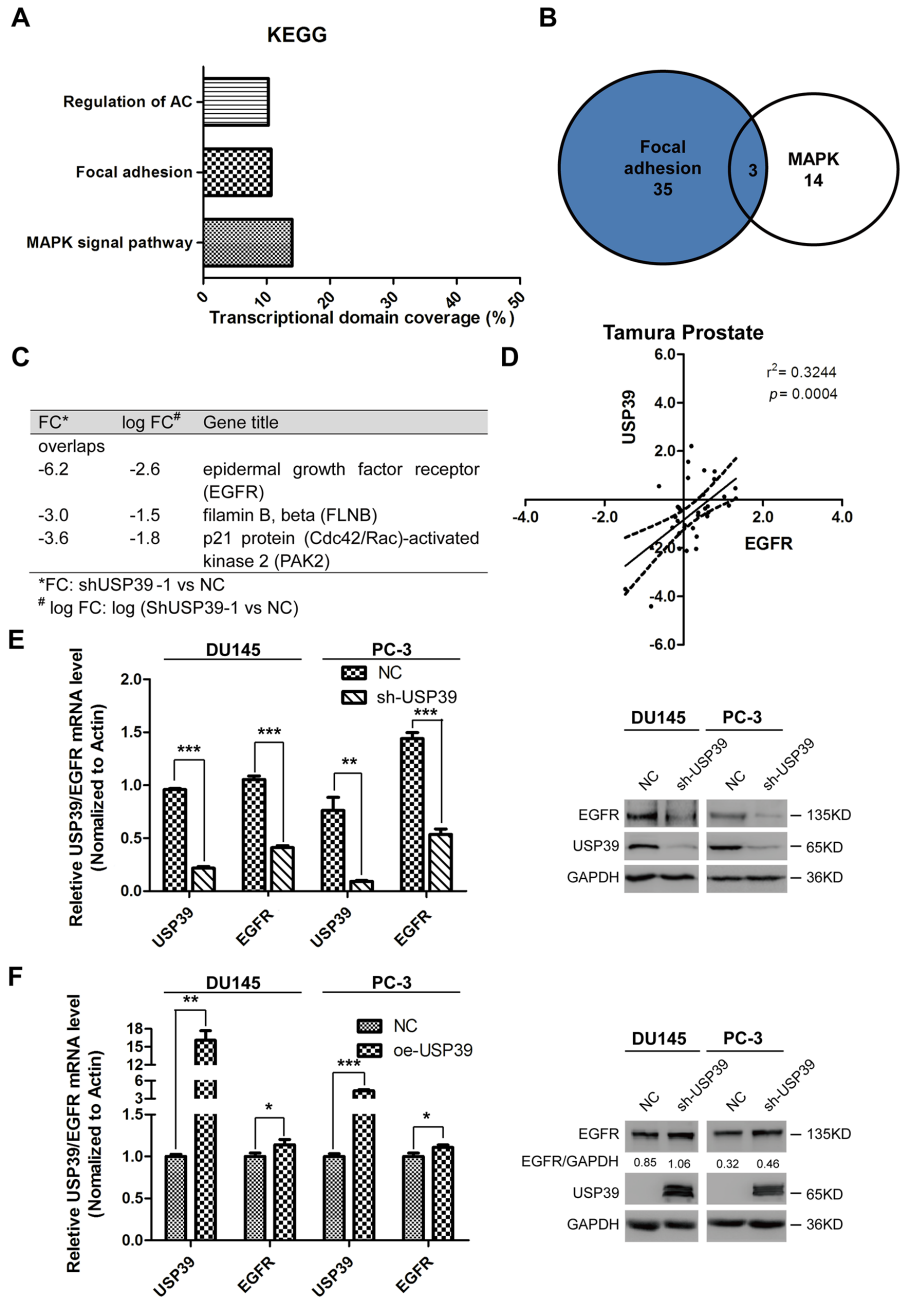
EGFR is a downstream target of USP39 **A, B.** and **C.** KEGG pathway analysis of Microarray results after USP39 knockdown. **D.** Correlation analysis using Tamura Prostate dataset (Oncomine). **E.** QRT-PCR and Western blot analysis of EGFR expression after USP39 knockdown in DU145 and PC-3 cells (DU145 : USP39, *P*=0.0000; EGFR,*P*=0.0001; PC-3: USP39, *P*=0.0048; EGFR, *P*=0.00010). **F.** QRT-PCR and Western blot analysis of EGFR expression after ectopic expression of USP39 in DU145 and PC-3 cells (DU145: USP39, *P*=0.0038; EGFR, *P*=0.0426; PC-3: USP39, *P*=0.0008; EGFR,*P*=0.0258).

To study the downstream signaling pathway, PC-3 cells were transduced with shUSP39 and subjected to microarray analysis. KEGG pathway analysis suggested that MAPK, focal adhesion and regulation of actin cytoskeleton pathways were first three most significant pathways (Figure 5A). EGFR showed an importance as was comprised by both the MAPK and focal adhesion pathways, and had the highest variation ratio(Figure 5B and 5C). Through ONCOMINE data-mining, we identified that USP39 level was positively correlated with EGFR expression (r^2^=0.3244, *P*=0.0004) in Tamura prostate dataset (Figure 5D). Knockdown of USP39 downregulated EGFR expression in PC-3 and DU145 cells in mRNA (*P*<0.0001 and *P*<0.0001, respectively) (Figure 5E) and protein levels. Consistently, overexpression of USP39 upregulated EGFR expression both in both mRNA levels (PC-3:*P*=0.0258; DU145:*P*=0.0426) and protein levels (Figure 5F).

### USP39 is a pivotal regulator of EGFR pre-mRNA splicing and transcription elongation

**Figure 6:**
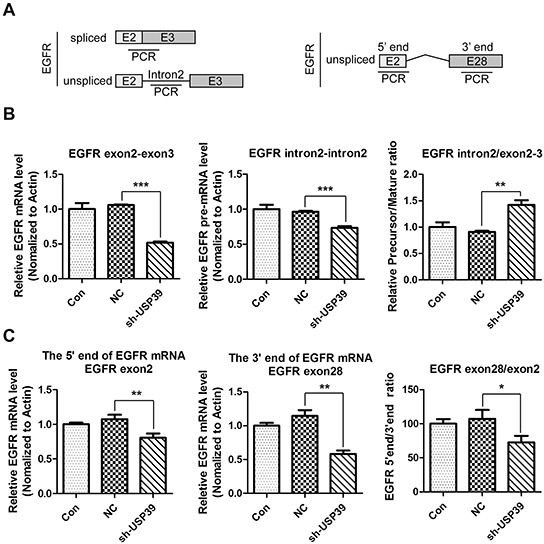
USP39 regulates pre-mRNA splicing and transcription elongation of EGFR **A.** Four pairs of primers were designed as illustrated to detect intron2, exon2-exon3 junction, exon2 and exon28. **B.** QRT-PCR was conducted to detect each fragment (middle panel: EGFR-intron2, *P*=0.0004;left panel: EGFR-exon2-exon3, *P*<0.0001). The ratio of pre-mRNA to mature mRNA in each group was presented (*P*=0.0078). **C.** QRT-PCR was used to analyze the expression levels of exon2 (left panel) and exon28 (middle panel). The ratio of exon28 to exon2 is presented in the right panel(EGFR-Exon2,*P*=0.0069;EGFR-Exon28, *P*=0.0015; exon28/exon2, *P*=0.0297).

Knowing that USP39 is an important regulator of pre-mRNA machinery, we further investigated whether USP39 was required for splicing of EGFR pre-mRNA. QRT-PCR were performed using intron2 and exon2-exon3 junction-specific primers to distinguish spliced and unspliced mRNA levels, and the splicing efficiency was calculated by the ratio of spliced to unspliced transcripts (Figure 6A). As shown in Figure 6B (left panel and middle panel), both pre-mRNA and mature mRNA were downregulated upon USP39 silencing (*P*<0.0001 and *P*=0.004). Nevertheless, as the mature EGFR mRNA had a higher reduction rate, the splicing efficiency of EGFR pre-mRNA was reduced and the precursor was accumulated (*P*=0.0078) (Figure 6B right panel).

To analyze whether USP39 could affect the expression of the 3′ end of EGFR, we designed two pairs of primers on exon2 and exon28 (Figure 6A). The results demonstrated that the level of the 3′-end of EGFR decreased (*P*=0.0015) (Figure 6C, middle panel) more sharply than that of the 5′-end (*P*=0.0069) (Figure 6C, left panel), and the ratio ofthe 3′-end to 5′-end levels was significant decreased (*P*=0.0297) (Figure 6C, right panel), implying that knockdown of USP39 might inhibit the transcription elongation of EGFR, and might produce unstable EGFR mRNA fragments lacking the 3′-UTR.

## DISCUSSION

So far, the clinical significance and downstream mechanisms of USP39 as a member of spliceosomes in PCa remains elusive. Immunohistochemical analysis and oncomine data-mining showed that USP39 was overexpressed in PCa tissues. The elevated expression of USP39 was positively correlated with Gleason score, and was an independent risk factor for BCR. USP39 was also upregulated in PCa cell lines compared with normal prostate cell RWPE-1, and especially in AR-negative PC-3 and DU145 cells. Knockdown of USP39 suppressed the proliferative ability of PC-3 and DU145 cells, causing G2/M arrest and inducing apoptosis of PCa cells by decreasing splicing and transcription elongation of EGFR mRNA. These findings demonstrated that USP39 is an important biomarker and putative oncogene in PCa.

USP39 is a well-defined component of splicesome, controlling pre-mRNA splicing of proto-oncogenes aurora B and RB1 [6, 8], implying that it has a growth-promoting function. However, whether this function is related to cancer formation has not been studied yet. This study identified EGFR as a target of USP39 in PCa, and that USP39 was positively correlated with EGFR mRNA expression in Tamura prostate dataset (ONCOMINE). Knockdown of USP39 suppressed the proliferation and cell cycle progression, and induced apoptosis, accompanied by the reduction of EGFR in both mRNA and protein levels in PC-3 and DU145 cells. To verify whether the regulatory effect of USP39 was EGFR specific, ectopic expression of USP39 were performed in PC-3 and DU145 cells, which showed that overexpressed USP39 upregulated EGFR mRNA and protein levels, indicating that EGFR is a downstream target of USP39. EGFR expression has been proved to be a risk factor for PCa [18–21], acting as a proto-oncogene. Here we identified that USP39 promoted tumorigenesis of PCa through activating EGFR pathway.

It is known that pre-mRNA is subjected to mRNA processing in eukaryotes including capping, polyadenylation, and splicing [22]. We hypothesized that USP39 could up-regulate EGFR by increasing the pre-mRNA splicing. To verify this, we designed two pairs of primers, recognizing only intron2 on mRNA precursor or exon2-exon3 junction on mature mRNA. As expected, intron 2/exon2-exon3 ratio was upregulated, indicating the accumulation of EGFR mRNA precursor and suppression of pre-mRNA splicing. However, the level of EGFR mRNA precursor (intron 2) was also reduced upon USP39 silencing, implying that USP39 might be also involved in the processes ahead of pre-mRNA splicing. To further clarify this surmise, we detected the levels of exon2 (5′-end) and exon28 (3′-end). The results showed that the expression of both exon2 and exon28 were downregulated, and the reduction rate of exon28 was higher than that of exon2, suggesting that USP39 was also involved in transcription elongation. A lack of 3′-UTR may reduce the stability of mRNA, which explains why exon2 was decreased. Transcription elongation arrest is considered as a checkpoint mechanism in preventing the accumulation of pre-mRNA due to splicing deficiency. U2snRNPis a main component of splicesome, and participates in both maturation of EGFR pre-mRNA and dowregulation of EGFR 3′-UTR [22]. USP39 cloud recruit the complex of U4/U6. U5 tri-snRNP to form the complex A (U1-U2snRNPs compound) [7]. Then the complex B (U1-U2-U4/U6.U5tri-snRNP compound) could be produced to remove the introns. Probably, USP39 regulates the expression of EGFR 3′-end via U2snRNP.

Knockdown of USP39 has been reported to inhibit the growth of hepatocellular carcinoma (HCC), breast cancer and medullary thyroid carcinoma cells [16, 23–25]. A sumosylated USP39 has been shown to promote the proliferation of PC-3 and LNcap cells [17]. Nevertheless, the clinical significance of how USP39 is related to PCa has not been elucidated. In this study, we performed immunohistochemical staining and ONCOMINE database analysis to investigate the correlation and the prognostic value of USP39 with PCa. It was found that USP39 was overexpressed in PCa tissues. Through IHC (Figure 1B) and ONCOMINE dataset analysis (Figure 1E), we found that USP39 was expressed less in the lower gleason score 7 compared with the higher scores (*P*=0.042 and *P*=0.013). Therefore, elevated USP39 was positively correlated with Gleason score and negatively correlated with BCR (Figure 2A and 2B). It can be functioned as an independent risk factor in univarate analysis and a hazard factor in multivariate analysis. These observations are consistent with the previous finding that USP39 was overexpressed in HCC and breast cancer tissues [16, 24]. It was found in our Kaplan-Meier survival analysis that high USP39 expression predicted a shorter BCR free duration. Also, we found that in the cohort of patients with a Gleason score ≤7, USP39 expression could work as prediction factor even when Gleason score loses its prediction ability.

In summary, this study indicated that USP39 is a biomarker for BCR of PCa patients. Knockdown of USP39 inhibits malignant transformation of PCa through interrupting the transcription elongation, maturation and stability of EGFR mRNA.

## MATERIALS AND METHODS

### Clinical specimens

A total of 45 prostate cancer specimens were from radical prostatectomy at the department of urological surgery of Changzheng Hospital (shanghai, China) between March 2012 and January 2014. The patients were confirmed by preoperative CT and MRI examinations of the pelvic cavity that showed no sign of metastasis, and by radionuclide bone scan (RBS) that showed no bone metastasis. 8 out of 45 had only cancer tissues, whereas other 37 had paired normal tissue specimens. Therefore, only 37 pairs of specimens were subjected to the analysis of the differential expressions of USP39 between prostate cancer tissues and paired normal prostate tissues. All specimens were evaluated histologically by a pathologist who was blind to the clinical data of the patients. All specimens were fixed in formalin, embedded in paraffin, HE stained and evaluated histologically by a pathologist who was blind to the clinical data of the patients. The information on 45 prostate cancer patients were provided as Data Set. The study was approved by the ethics committee of the Second Military Medical University (Shanghai, China).

### Cell culture

RWPE, DU145, PC-3, LNCaP and 22Rv1 cell lines (Cell Bank of the Chinese Academy of Sciences, Shanghai, China) were cultured as described previously [26]. RWPE cells were cultured in Keratinocyte serum-free medium supplemented with epidermal growth factor (EGF), bovine pituitary extract (BPE), 10% fetal bovine serum (FBS) and 1% glutamine PenStrep (Gibico). DU145 and PC-3 cells were cultured in Ham's F-12 Nutrient Mixture (Gibico). LNCaP and 22Rv1 were cultured in RPMI-1640 (Hyclone).

### Plasmid, shRNA, and transfection

ShRNA sequences targeting USP39 (5′-GATTTGGAAGAGGCGAGATAA-3′) was inserted into lentivirus vector pFL-GFP (Hollybio. Inc. Shanghai, China). The USP39 ORF was subcloned from total RNA ofDU145 cells and inserted into pTAL7A-1xFlag-1xHA vector (Hollybio. Inc. Shanghai, China). Lentivirus was produced in 293T cells by the Calcium phosphate transfection method. The knockdown efficiency was evaluated by real-time PCR and Western blotting. This construct was confirmed by DNA sequencing.

### MTT assay

Cell viability were assessed by 3-(4, 5-dimethylthiazol-2-yl)-2, 5-diphenyltetrazolium bromide (MMT) assay. Cells stably transduced after USP39 knockdown were recultured in 96-well platesat a density of 2000 cells/ well for five point-in-time (day 1, 2, 3, 4 and 5). After an additional 4-hincubation at 37°C, the reaction was terminated by acidic isopropanol solution (10 % SDS, 5 % isopropanol and 0.0 1 mol/l HCl). After 0.5 hr, the absorbance at 595 nm was measured to evaluate cell viability. All samples had three repetitions accordingly.

### Flow cytometric analysis of apoptosis

After 120-h transduced, cellswere trypsinized, centrifuged, washed with ice-cold PBS, and fixed in 75% ethanol overnight and incubated in 500μLpropidium iodide (PI) staining solution (PI dye and RNase A) at 37°C. Cell cycle distribution was finally analyzed by flow cytometry (BD Biosciences, USA). All samples had three repetitions.

### Plate colony formation assay

After 72-h infection, cells were trypsinized, suspended, counted, plated in a 6-well plate at a density of 400 cells/well and incubated at 37°C for 14 days. The culture medium was changed every 3 days. The cell colonies were fixed in4% paraformaldehyde for 30 min and stained with GIEMSA for 20min. Each cell colony was counted under a light microscope and photographed using a digital camera. All samples had three repetitions.

### Western blotting analysis

Cells were lysed with lysis buffer (50 mMTris-HCl, pH 7.4, 150mM DTT, 0.1% SDS, 10% glycerol, and protease inhibitor (cocktail), phosphatase inhibitor). Cell lysates were centrifuged at 12,000g for 15min, and proteins in the supernatants were quantified. Protein extracts were mixed with loading buffer and run on a 10% SDS-polyacrylamide gel, electrophoresed, and transferred to a nitrocellulose membrane (Amersham Pharmacia Biotech, Buckinghamshire, UK). The membrane was blocked with 5% nonfat milk in TBS containing 0.05% Tween 20 for 1 h at room temperature. The membrane was incubated with the primary antibodies for 2h, soaked, and incubated for 1 h with HRP-conjugated secondary antibodies (cell signaling technology). Chemiluminescence of the membrane was detected using a Western blotting detection system (Tanon).

### Quantitative real-time PCR analysis

Total RNAs were isolated using Trizol reagents (Takara) and were subjected to DNaseI treatment prior to reverse transcription using random hexamers and M-MLV-RTase (Takara). The resulting cDNAs were subjected to qRT-PCR with the indicated primer sets. The primer sequences were used: for USP39, 5′-GCCAGCAGAAGAAAAAGAGC-3′ (forward) and 5′-GCCATTGAACTTAGCCAGGA-3′ (reverse); for EGFR-exon2-exon3, 5′-CAAGGCACGAGTAACAAG-3′ (forward) and 5′-GGCAATGAGGACATAACC-3′ (reverse); for EGFR-intron2, 5′-GGAGAGTGTTGAA CCCCGTGAG-3′ (forward) and 5′-CGCCCAGTTGAA CCCTAATGC-3′ (reverse); for EGFR-exon2, 5′-CCAAGG CACGAGTAACAAGC-3′ (forward) and 5′-AAAGGG TGTAACGCAACTA-3′ (reverse); for EGFR-exon28, 5′-GACTGA CTTGTTTGTCTTCCATTCC-3′ (forward) and 5′-GCGG TGCTATCCTTAGGTATTCC-3′ (reverse); for β-actin, 5′-ATCGTGCGTGACATTAAGGAG-3′ (forward) and 5′-AGGAAGGAAGGCTGGAAGAG-3′ (reverse). Values were normalized to those of Actin.

### Microarry screening

Total RNA was extracted from PC-3 cells using Trizol reagents according to the manufacturer's instructions. Total RNA was quantified by the BioTek Epoch system (America). Microarray analysis was provided by OE Biotech. Co., Ltd (Shanghai, China). Fold change was used to identify the differential expressions of genes. A fold change>=2.8 was applied for the threshold set of up-and down-regulated genes. The roles of these differentially expressed mRNAs were then determined by GO analysis and KEGG analysis.

### Immunohistochemical staining

Paraffin blocks of all tissues were cut into 5-μm thick sections and mounted on polylysine-coated glass slides. The sections were dewaxed in xylene, soakedwith100%, 90%, 80% and 70% ethanol and ddH_2_O, incubated in 1:100 dilution of rabbit anti-USP39 antibody (Proteintech, UK) at 4°C overnight, and then with biotinylated goat anti-rabbit serum and streptavidin-peroxidase conjugate for 15min. Finally, the 3′, 3′-diaminobenzi dine was used for color reaction of the sections.

The staining site and intensity were evaluated by light microscopy with two independent pathologists. According to the percentage of positively stained cells and the distribution of staining intensity, they were classified into four grades: negative 0%, weak 0%∼20, medium 20%∼60%, and strong 60%∼100%.

### Oncomine database analysis

A set of microarray data in Glinsky and Tamura Prostate were analyzed with the Oncomine Compendium of Expression Array data (www.oncomine.org). The following search terms were used: “USP39”, “Prostate Cancer” and “mRNA”. A total of 31 datasets were included according to primary screening. Then, further screening was carried out according to requirement of our study, such as “Cancer vs. Normal Analysis” and “Recurrence Status”. In Glinsky Prostate, 79 prostate tissue samples were collected on Affymetrix U133 Plus 2.0 microarrays with the tissue type of PCa. The different Gleason grade category and the recurrence status were analyzed by different expression levels of USP39. In Lapointe and Vanaja Prostate dataset, samples were analyzed in the same way. Each dataset was processed by GraphPad Prism 5.

### Statistical analysis

Differences in categorical variables between two groups were analyzed by Chi-square test. Clinicopathologic parameters between the expressions of USP39 were evaluated by Kruskalwallis test, and correlations between them were analyzed by Spearman test. All statistical analyses were conducted using SPSS 21.0 software package (SPSS Inc, Chicago, IL, USA), and *P*<0.05 was considered statistically significant.

## SUPPLEMENTARY FIGURE




