# Supplementary material for: Overexpression of USP39 predicts poor prognosis and promotes tumorigenesis of prostate cancer via promoting EGFR mRNA maturation and transcription elongation
Source: Oncotarget. 2016 Mar 3;7(16):22016–30. doi: 10.18632/oncotarget.7882 (PMC5008341; doi:10.18632/oncotarget.7882)
Supplement: Supplementary file 1 [file oncotarget-07-22016-s001.pdf]

SUPPLEMENTARY FIGURE

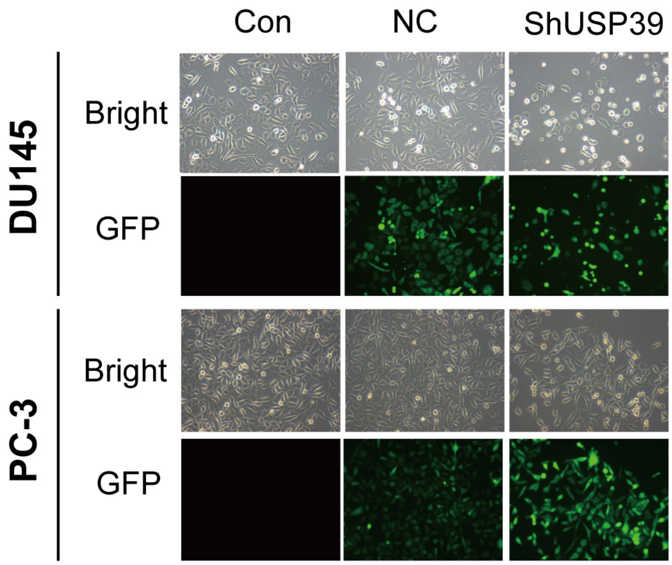

Supplementary Figure S1: Lentivirus-mediated knockdown of USP39 in PC-3 and DU145. The lentivirus was successfully transfected into PC-3 and DU145 cells. The result was measured in in bright and GFP (magnification ×100).
